# Supplementary material for: Vector Competence of Mosquitoes from Germany for Sindbis Virus
Source: Viruses. 2022 Nov 26;14(12):2644. doi: 10.3390/v14122644 (PMC9785343; doi:10.3390/v14122644)
Supplement: Supplementary file 1 [file viruses-14-02644-s001.zip › viruses-2052715-supplementary.pdf]

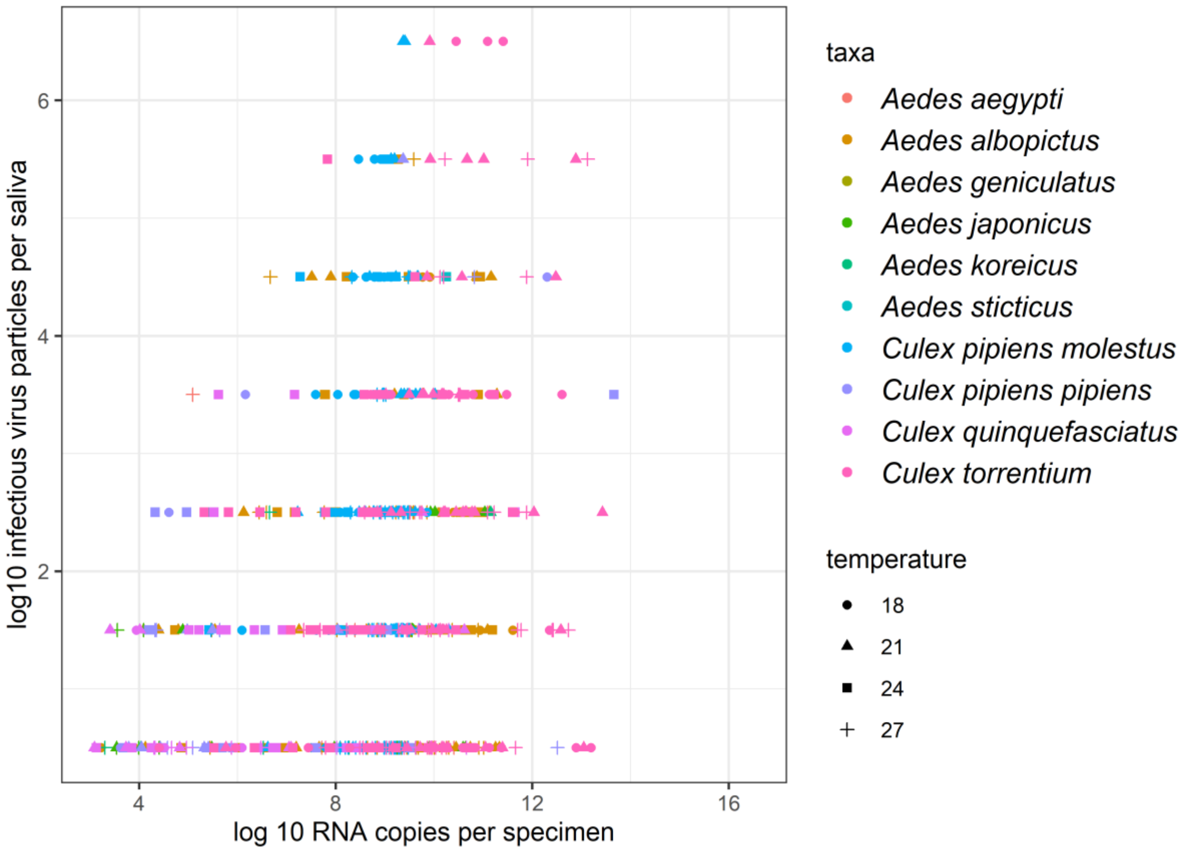

Additional File 1, Figure S1. Relationship between body titers and sal iva titers per specimen.

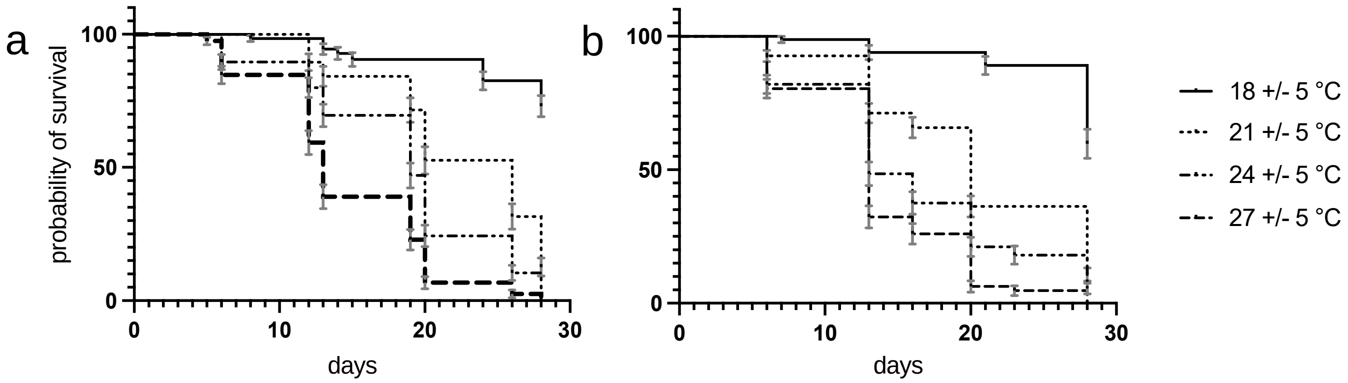

Additional file 2, Figure S2. Survival of (a) uninfected and (b) SINV infected *Cx. pip.* biotype *molestus* mosquitoes depending on incubation temperature for 28 days after blood feed. Number of alive mosquitoes was counted once a week. Significance was tested by log-rank (Mantel-Cox) test, standard error is shown in the graph (grey). (a) 18 +/- 5 °C n = 126; 21 +/- 5 °C n = 95; 24 +/- 5 °C n = 115; 27 +/- 5 °C n = 118. 18 +/- 5 °C vs. 21 +/- 5 °C: df = 1, X^2 = 25.75. 18 +/- 5 °C vs. 24 +/- 5 °C: df = 1, X^2 = 167.6. 18 +/- 5 °C vs. 27 +/- 5 °C: df = 1, X^2 = 209.9. 21 +/- 5 °C vs. 24 +/- 5 °C: df = 1, X^2 = 25.75. 21 +/- 5 °C vs. 27 +/- 5 °C: df = 1, X^2 = 73.33. 24 +/- 5 °C vs. 27 +/- 5 °C: df = 1, X^2 = 22.83. All temperature profiles are statistically significant different to each other (P = < 0.0001).
(b) 18 +/- 5 °C n = 119; 21 +/- 5 °C n = 149; 24 +/- 5 °C n = 128; 27 +/- 5 °C n = 127. 18 +/- 5 °C vs. 21 +/- 5 °C: df = 1, X^2 = 72.06. 18 +/- 5 °C vs. 24 +/- 5 °C: df = 1, X^2 = 107.8. 18 +/- 5 °C vs. 27 +/- 5 °C: df = 1, X^2 = 162.7. 21 +/- 5 °C vs. 24 +/- 5 °C: df = 1, X^2 = 18.18. 21 +/- 5 °C vs. 27 +/- 5 °C: df = 1, X^2 = 61.31. 24 +/- 5 °C vs. 27 +/- 5 °C: df = 1, X^2 = 10.10. All temperature profiles of SINV infected mosquitoes are statistically significant different to each other (P = < 0.0001), except 27 +/- 5 °C vs. 24 +/- 5 °C has a lower P-value of 0.0015.

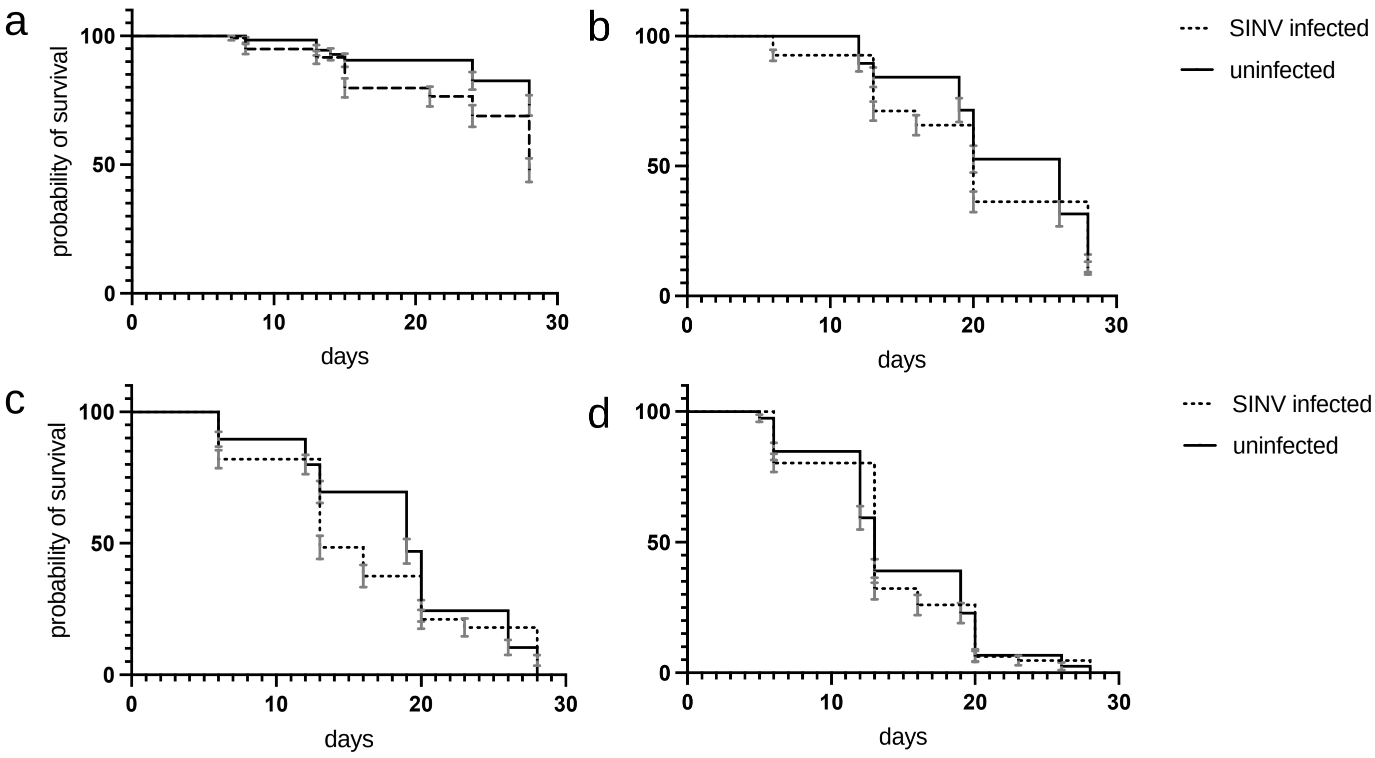

Additional file 3, Figure S3. Comparison of the survival of uninfected and SINV infected *Cx. pip.* biotype *molestus* mosquitoes incubated for 28 days at four different temperatures. Number of alive mosquitoes was counted once a week. Significance was tested by log-rank (Mantel-Cox) test, standard error is shown in the graph (grey). (a) 18 +/- 5 °C, uninfected n = 126 and SINV-infected n = 119, df = 1, X^2 = 15.51, P = < 0.0001. (b) 21 +/- 5 °C, uninfected n = 95 and SINV-infected n = 149, df = 1, X^2 = 0.316, P = 0.574. (c) 24 +/- 5 °C, uninfected n = 115 and SINV-infected n = 128, df = 1, X^2 = 0.1157, P = 0.7337. (d) 27 +/- 5 °C, uninfected n = 118 and SINV-infected n = 127, df = 1, X^2 = 1.063, P = 0.3026.
